# Concurrent nonindependent fixed‐ratio schedules of alcohol self‐administration: Effects of schedule size on choice

**DOI:** 10.1002/jeab.215

**Published:** 2016-07-12

**Authors:** Richard A. Meisch, Thomas H. Gomez

**Affiliations:** ^1^ The University of Texas Health Science Center at Houston

**Keywords:** choice, alcohol self‐administration, nonindependent fixed‐ratio schedules, schedule size, preference reversal, changeover responses, mouth‐contact response, rhesus monkeys

## Abstract

Choice behavior was studied under concurrent nonindependent fixed‐ratio fixed‐ratio (nFR) schedules of reinforcement, as these schedules result in frequent changeover responses. With these schedules, responses on either operandum count toward the completion of the ratio requirements of both schedules. Five monkeys were subjects, and two pairs of liquid reinforcers were concurrently available: 16% (w/v) and 0% ethanol or 16% and 8% ethanol. For each pair of reinforcers, the nFR sizes were systematically altered across sessions while keeping the schedule size equal for both liquids. Responding varied as a function of reinforcer pair and nFR size. With the 16% and 0% pair, higher response rates were maintained by 16% and were an inverted U‐shape function of nFR size. With 16% and 8%, a greater number of responses initially occurred on the schedule that delivered 8% ethanol. However, as nFR size increased, preference reversed such that responses that delivered 16% ethanol were greater. When the nFR size was subsequently decreased, preference reverted back to 8%. Number of responses emitted per delivery was a dependent variable and, in behavioral economic terms, was the price paid for each liquid delivery. With 16% and 0%, changeover responses initially increased and then decreased as schedule size became larger. In contrast, with the 16% and 8% pair, changeover responses increased directly with schedule size. Responding under nFR schedules is sensitive to differences in reinforcer magnitude and demonstrates that relative reinforcing effects can change as a function of schedule size.

A central topic in the study of behavior is choice. Most studies of choice employ concurrent interval schedules, as changeover responses are more frequent with interval schedules than with concurrent ratio schedules due to the probability of reinforcer availability increasing with the passage of time. However, with nonindependent concurrent fixed‐ratio fixed‐ratio (nFR) schedules, the probability of a reinforcer becoming available on the opposite schedule increases as a function of the number of responses. Thus, these schedules are also suitable for the study of choice as responding is distributed across both schedules.

Nonindependent concurrent fixed‐ratio fixed‐ratio (nFR) schedules are of interest, as they are the formal and functional equivalent of concurrent interval schedules, differing only in that delivery of reinforcers is contingent upon the number of responses instead of the passage of time (MacDonall, 1988). However, few studies have been published concerning these schedules. Such schedules are useful in studies of choice as they increase the number of changeover responses, do not result in exclusive preferences, and provide the dependent variable of responses per delivery as a continuous rather than step‐wise measure (Meisch & Gomez, 2013; Meisch & Spiga, 1998).

Under nonindependent concurrent ratio schedules, responses on either the left or right operandum count toward completion of both left and right ratio schedules. When a reinforcer is earned on the side opposite from that on which responding is occurring, the delivery of that reinforcer is held until the animal switches and makes a response to the side where the reinforcer is available. Availability of a reinforcer on the opposite side does not alter the contingencies on the side where responding is occurring. These schedules are similar to concurrent variable‐interval (VI) and fixed‐interval (FI) schedules, in that progress toward schedule completion occurs simultaneously on both schedules. With concurrent VI or FI schedules, when a schedule requirement has been met on the concurrently available schedule, a single reinforcer remains available on that schedule until a response is made on that side and the reinforcer is collected. Correspondingly, under a nonindependent concurrent FR schedule, completion of three FR requirements on one side, for example, would make only one reinforcer available on the opposite side.

Nonindependent ratio schedules were first described by Shull and Pliskoff (1971), and were further explored by MacDonall (1988, 1998, 1999). With rats as subjects and food as the reinforcer, matching (relative response rate being proportional to relative relative‐reinforcer‐delivery rate) was found with concurrent nonindependent variable‐ratio variable‐ratio (VR VR) schedules (MacDonall, 1988). Matching was also obtained under nonindependent VR VR schedules with rhesus monkeys as subjects and orally delivered pentobarbital as the reinforcer (Meisch & Spiga, 1998). Four objectives of the present study are described next, in the context of the special characteristics of these nonindependent schedules.

With nonindependent ratio schedules, each changeover response (defined as the first response made on the operandum opposite to that from the previous response) decreases the required number of responses for both reinforcers. Thus, responses per delivery become a continuous, rather than step‐wise dependent variable (Meisch & Gomez, 2013), and the frequency of changeover responses increases (Shull & Pliskoff, 1971; Meisch & Gomez, 2013). These features are important in studies of choice because the probability of consumption of reinforcers from each side is increased, and responses per delivery can be used as a graded measure of choice (Meisch & Gomez, 2013). Nonindependent ratio schedules may also aid studies of behavioral economics because the subject's responding can adjust the relative cost of two choices. In contrast to *nonindependent* FR schedules, use of *independent* concurrent FR FR schedules often produces an exclusive preference for one of the two reinforcement schedules (Shull & Pliskoff, 1971). Prior studies have not systematically examined identical increases and decreases in the size of both nonindependent ratio schedules. Thus, a first objective of the present study was to determine the effects of varying the ratio size. A second objective was to also vary the magnitude of the reinforcer concurrently available with an unchanging 16%‐ethanol solution: 16% and 0% ethanol compared to 16% and 8% ethanol. In earlier studies, food had been used as the reinforcer, and rats and pigeons had served as subjects (MacDonall, 1988, 1998, 1999; Shull & Pliskoff, 1971). In addition, the size of the concurrent schedules usually was varied, but the size of the food reinforcer was held constant. In the present study the size of both concurrent schedules was the same within pairs; however, the magnitude of the ethanol reinforcer available under one of the schedules varied. Relative persistence of responding is a measure of relative reinforcing effects (Meisch, 2000). It is calculated by dividing rates of behavior at a higher schedule size by rates at a lower baseline schedule size (usually FR 1). The results correspond closely with a number of measures of preference, although not always with progressive‐ratio performance (Gomez & Meisch, 2003). Relative persistence is a measure not based on choice, although it can be combined with measures of choice. In prior studies of relative persistence in our laboratory, FR or signaled DRL schedules were used. With these schedules there is a close correspondence between the number of responses and the number of reinforcer deliveries, but it has not been clear whether one measure is more important than the other. Nonindependent schedules do not necessarily produce a fixed relation between responses and reinforcer deliveries. Thus, a third objective of the present study was to evaluate the equivalence, or difference, of numbers of responses versus numbers of deliveries as measures of relative persistence. A fourth objective was to determine if relative‐persistence measures made with *nonindependent* schedules produce findings similar to those seen in earlier studies with *independent* schedules (Meisch, 2000).

## Materials and Methods

### Subjects

The subjects were five adult male rhesus monkeys (*Macaca mulatta*); at the beginning of the study, ages in years were: Crash 16, JoJo 22, Lucas 7, Raja 16, and Tango 25. All of the monkeys had more than a 6‐year history of oral drug self‐administration, except for subject Lucas who had recently completed a procedure to establish ethanol as a reinforcer. To establish ethanol‐reinforced behavior, Lucas was given a gradually increasing series of ethanol concentrations: 2, 4, 5.7, 8, 11.3 and 16% (w/v) across daily 3‐hr sessions. Subject Crash had self‐administration experience with ethanol, methadone, and cocaine; JoJo with ethanol and cocaine; Raja with ethanol and methadone; and Tango with ethanol, cocaine, and methadone. At the beginning of the study their weights (kg) were: Crash, 9.1; JoJo, 12.3; Lucas, 7.8; Raja, 9.6; and Tango, 11.8. Subjects were housed individually in their chambers (described below) in a climate‐controlled room (22.8°C) in which a 12‐h light and dark cycle was in effect (lights on at 0700). Subjects were maintained at 85 to 90% of their normal weights through daily feeding with a measured amount of commercially available chow (Lab Diet® High Protein Monkey Diet # 5045, PMI Nutrition International, Brentwood, MO), plus fresh fruit and vegetables. The amount of food was sufficient to maintain stable body weights, and the monkeys’ health and appearance were good. Food restriction has been shown to enhance drug self‐administration (Carroll & Meisch, 1984) and reinforcing effects (Carr, 2002; Kliner & Meisch, 1989). Food restriction also prevents obesity (Meisch & Lemaire, 1988), and increases median life span and general health (Kemnitz, 2011; Mattison, Roth, Lane, & Ingram, 2007). Animal care was in accordance with the guidelines of the Institute of Laboratory Animal Resources, Commission on Life Sciences, National Research Council (2011), and the Institutional Animal Use and Care Committee of The University of Texas Health Science Center at Houston approved all procedures.

### Apparatus

The monkeys were individually housed for the duration of the study in stainless‐steel primate cages (Lab Products, Sanford, DE), which also served as the experimental chambers. Each chamber (76 x 102 x 81 cm) had three solid walls and one barred wall. A liquid‐delivery apparatus panel was attached to the outside of one side wall, and spouts and stimulus lights protruded into the cage through holes cut in that wall. On the back of the apparatus panel was a T‐shaped bar; the horizontal portion of this bar was elevated above the level of the spouts, and on each limb of the horizontal bar was fastened a stainless‐steel reservoir covered with a lid. Delivery of liquid occurred as a function of the pressure differential between the elevated reservoir and the spout, with liquids contained in each reservoir passing through polyethylene tubing to a solenoid‐operated valve at the rear of one of the two brass spouts. These spouts (1.2 cm outside diameter, 0.2 cm inside diameter) extended 2 cm into the cage, 64 cm above the floor and 15.5 cm either side of the midline. The spouts were embedded in Plexiglas® disks that covered the 7‐cm‐diameter holes in the cage wall through which they entered. At each spout, two 1.1‐W lights, one located 2.5 cm on either side of the spout and visible through the Plexiglas®, were aligned diagonally; these spout lights were capped with green translucent lenses. Another two 1.1‐W spout lights, one located 2.5 cm on either side of the spout, were aligned on the opposite diagonal and were capped with white translucent lenses. Thus, each spout was in the center of a square pattern of four spout lights, two green and two white. The electronic components for the drinkometer circuit were housed in an enclosure at the rear of the spout. A 2.5‐cm‐diameter cluster of green light‐emitting diodes was located 11.5 cm directly above each brass spout. The liquid‐delivery apparatus has been described extensively elsewhere (Gieske, 1978; Henningfield & Meisch, 1976). The programming of experimental events and the recording of behavior were accomplished with a Dell® computer, MED‐PC® software, and Med Associates Inc. interface equipment.

Each reinforced response (both within and between sessions) operated a spout's solenoid for a set duration, typically of 150 ms, allowing gravity‐fed delivery of approximately 0.65 ml of liquid. The exact time of solenoid operation was calibrated for each solenoid, and the duration was controlled by the MED‐PC® software, and Med Associates Inc. interface equipment. A break in lip contact during liquid delivery terminated solenoid operation, thus preventing spillage. The volume of each liquid consumed in a session was calculated by measuring the differences in the volume in each reservoir before and after each session. The average volume of liquid per delivery was calculated by dividing each volume consumed by the number of deliveries occurring on the corresponding side. To keep the average volume of liquid per delivery at approximately 0.65 ml, the duration of operation of a spout's solenoid was adjusted as necessary across sessions. In four prior studies blood ethanol levels were determined and the levels confirmed that monkeys consume the ethanol solutions with this procedure (Henningfield & Meisch, 1978; Macenski & Meisch, 1992; Meisch & Lemaire, 1991; Stewart, Bass, Wang, & Meisch, 1996).

### Procedure

#### Sessions

Experimental sessions were 3 h in length (from 1000 to 1300 h) and were conducted 7 days per week. The cluster of green light‐emitting diodes above each spout, which functioned as a discriminative stimulus, blinked at a rate of 10 Hz when liquid was available from a spout during sessions. Identical discriminative stimuli were used for both spouts to control for differential responding that might otherwise result from the presence of dissimilar exteroceptive visual stimuli. Each mouth contact with a spout completed a drinkometer circuit and illuminated the green‐lensed pair of spout lights for the duration of the response. Deliveries of liquids were contingent upon completion of a nonindependent fixed‐ratio schedule. The final response in the schedule requirement initiated the liquid flow of approximately 0.65 ml of the appropriate solution. To further reduce the possible differential responding that might be caused by a monkey's preference for a particular spout, the drug and vehicle (water), or pairs of drug doses, were alternated between spouts each session. Nonindependent concurrent fixed‐ratio fixed‐ratio (nFR) schedules of reinforcement were used. Under these schedules, mouth‐contact responses on either operandum (spout) counted toward completion of both ratio requirements. When a ratio requirement was completed on the opposite side from which responding was occurring, the reinforcer (delivery) was withheld until the monkey responded on that spout. Upon collection of the reinforcer the programmed fixed‐ratio value was reset for the spout where the collection occurred.

Monkeys were presented with concurrently available solutions of 16 and 0% (w/v) ethanol solutions at FR 1. Subsequently, the nFR schedule was increased across sessions in the sequence 2, 4, 8, 16, 32, 64, and 128, with programmed fixed‐ratio values increasing identically for both operanda. After completing the condition at nFR 128, all monkeys were studied again at FR 1. Subsequently, with 16 and 8% ethanol concurrently available, the nFR schedule was increased across sessions in the sequence 2, 4, 8, 16, 32, 64, and 128, again with programmed fixed‐ratio values increasing for both operanda: nFR size was increased for each monkey across blocks of six sessions of stable behavior, until responding began to decline at a specific nFR value (which varied across subjects), at which point no further increases in the nFR value were made. The highest ratio value tested was nFR 256 for two monkeys, nFR 128 for two monkeys, and nFR 64 for one monkey. After completion of the highest nFR value at 16 and 8%, the sequence was reversed, and the nFR value was decreased across blocks of sessions to nFR 1. All conditions were studied for at least six sessions, until behavior was stable. Stability was defined as the absence of appreciable increasing or decreasing trends in the number of responses per session of either available liquid across six consecutive sessions. For each condition, the dependent measures for the last six sessions of stable behavior are expressed as the mean ± SD (standard deviation).

#### Between sessions

A timeout period was in effect during the hour immediately before each session (0900 to 1000 h). During this timeout period the number of water deliveries and the volume of water consumed since the last experimental session were recorded and liquids appropriate for the next session were placed in the two reservoirs. Each liquid‐delivery system was primed to ensure that the appropriate solution was present from a spout on the first delivery of an experimental session. Liquid volumes were measured after flushing to obtain the exact volume in the reservoirs at each session's onset. For 1 h immediately following the session (1300 to 1400 h) another timeout period was in effect, during which data from the session were recorded and water was placed into one reservoir and flushed through the tubing to the spout. Water was then available under an FR 1 schedule from one spout from 1400 h until 0900 h of the next day. The spout from which water was available between sessions alternated every other day. Between sessions, water availability was signaled by steady illumination of the appropriate cluster of green light‐emitting diodes above a spout, and each mouth contact was signaled by illumination of the white‐lensed pair of spout lights. At 1400 h the monkeys’ maintenance feeding was placed in the food hopper attached to the cage.

##### *Drugs*

Ethanol solutions were made from dilutions of 95% v/v ethanol approximately 20 h before the start of the sessions. Ethanol solutions were at room temperature at the start of the session. Dose was varied by changing the ethanol concentration.

## Results

**Figure 1:**
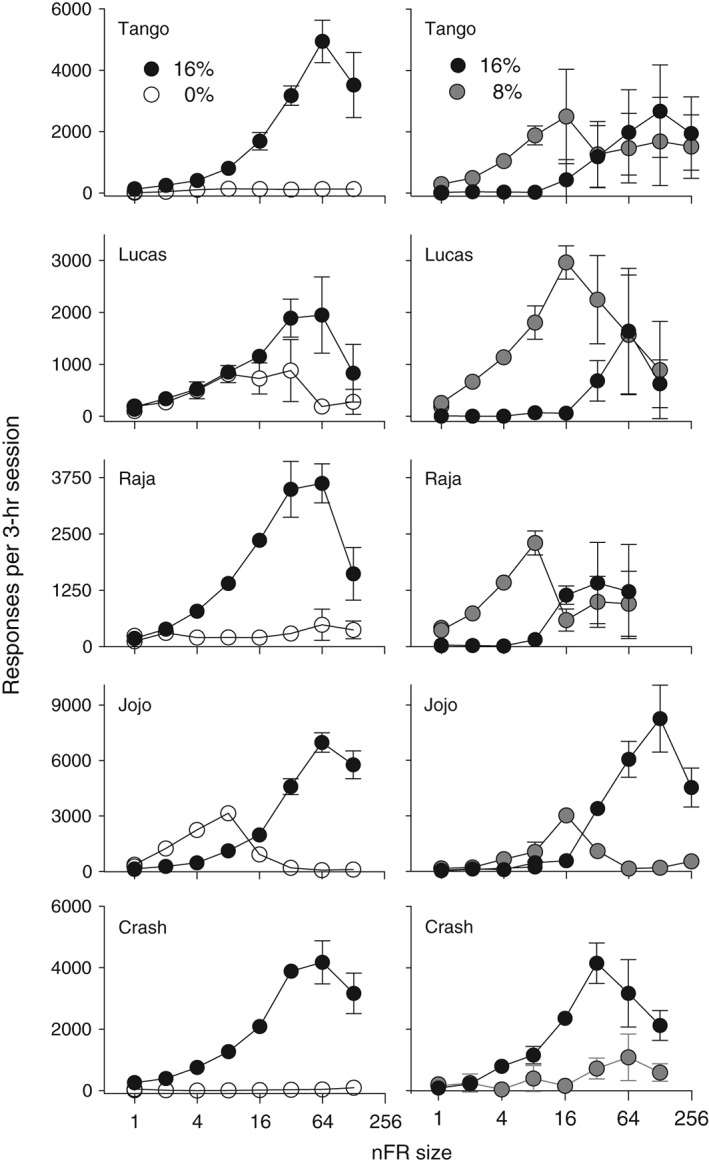
Responses are shown as a function of nFR schedule size for five monkeys. Panels on the left are for 16 and 0% ethanol (w/v) and panels on the right are for 16 and 8%. Each point is a mean of six consecutive sessions of stable behavior. Disconnected points at FR 1 represent redetermined values. Brackets denote the standard deviation (SD); absence of brackets at a point indicates the SD fell within the area occupied by the point. Note the different ordinate scales for each monkey.

Sixteen‐percent ethanol served as a reinforcer for all monkeys, as its response‐contingent delivery maintained far greater response rates than did the water vehicle (Fig. 1, left panels). For ethanol‐maintained behavior, responding was an inverted U‐shaped function of schedule size. An unusual pattern was displayed by the monkey JoJo. At low schedule sizes water‐maintained responding exceeded that maintained by ethanol; however, preference reversed with increases in the nFR value. After completion of the series of increasing schedule sizes, FR‐1 points were redetermined, and the redetermined points were similar to the initial values at FR 1. At the initial FR 1 determination the ethanol intakes for the five monkeys were 1.2, 2.5, 2.0, 1.1, and 2.5 g of ethanol per kg of body weight per 3‐h session (calculated from volume consumed and body weight), and the redetermined values were 112, 79, 99, 84 and 93 percent of the initial values for Tango, Lucas, Raja, JoJo, and Crash, respectively. The highest rate of responding was at the beginning of the session.

**Figure 2:**
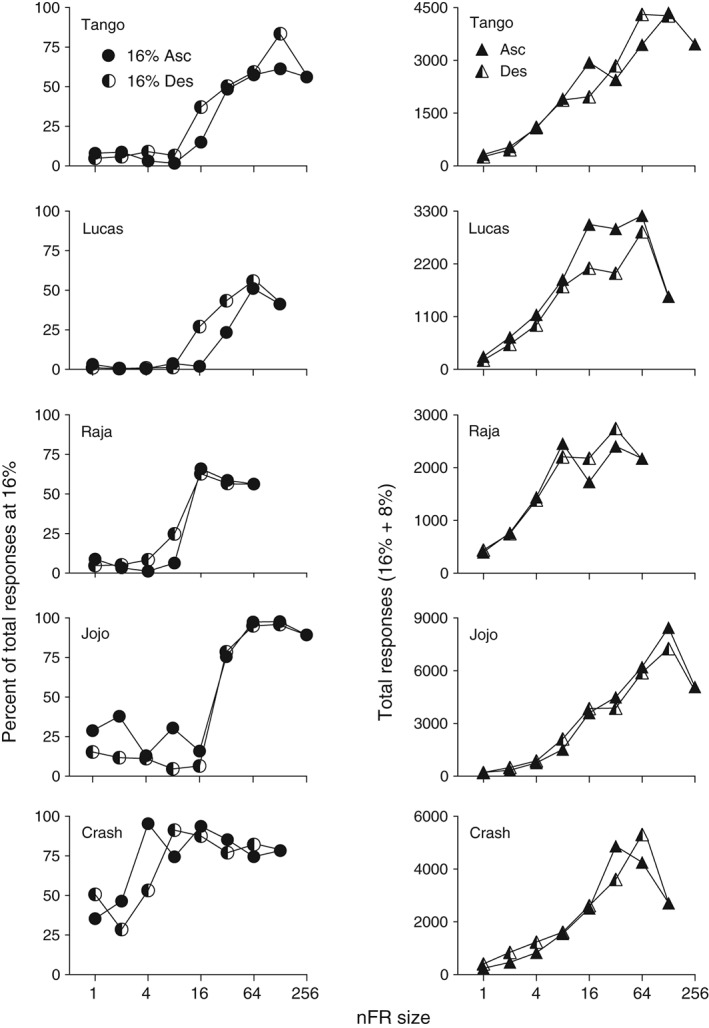
Percent of all responses (16% + 8%) comprised by responses on the spout that delivered 16% ethanol (w/v) (left panels), and total responses per session (16% + 8%) (right panels). Filled symbols represent means for the ascending series of nFR sizes; half‐filled symbols indicate values for the descending series. Each point is a mean of six consecutive sessions of stable behavior. Note the different ordinate scales for each monkey in the right column.

When 16 and 8% ethanol were concurrently present and schedule size was FR 1 all monkeys preferred 8% (Fig. 1, right panels; Fig. 2, left panels). As schedule size increased all monkeys showed an increase in responding maintained by 16% ethanol, two of the monkeys developed a clear preference for 16%, while two others showed a less obvious, but still present preference for 16%. Monkey Lucas, who showed the least increase in responding maintained by 16%, was also the least experienced with ethanol self‐administration. When the schedule size was reduced all monkeys reversed their preference from 16% to 8% (Fig. 2, left panels), and the results were similar to the ascending series of schedule sizes.

At 16 and 8% ethanol the differences in response rates on the concurrent nFR schedules at the higher schedule sizes were less than when the two liquids were 16 and 0%, an indication of more similar reinforcing effects when the two ethanol solutions were present. At higher nFR sizes, two monkeys (Lucas and Raja) developed a side preference that was not seen when the choice was between 16 and 0%. The side preference may reflect a closer degree of relative reinforcing effects at 16 and 8% than at 16 and 0%. The side preference accounts for the increased variability at nFR 64 and 128 for Lucas and at nFR 32 and 64 for Raja. The largest difference in responding maintained by 16% over the alternative liquid was usually at the next‐to‐the‐highest ratio value studied.

**Figure 3:**
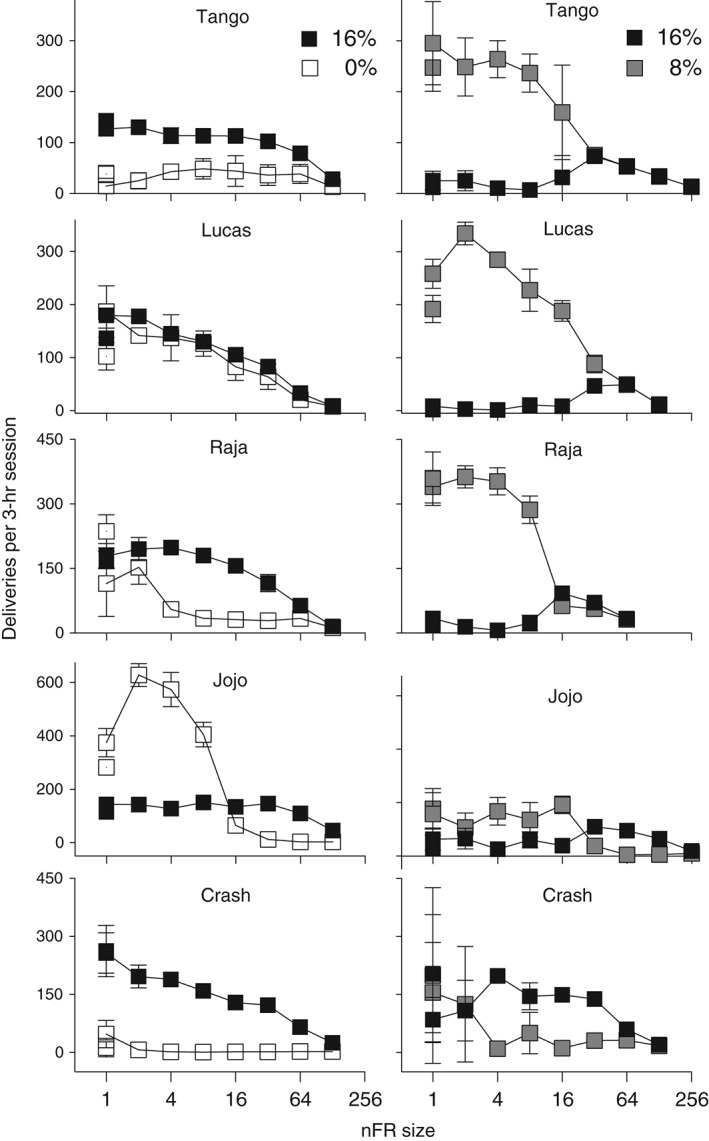
Liquid deliveries are shown as a function of nFR schedule size for five monkeys. Panels on the left are for 16 and 0% ethanol (w/v) and panels on the right are for 16 and 8%. Each point is a mean of six consecutive sessions of stable behavior. Disconnected points at FR 1 represent redetermined values. Brackets denote the standard deviation (SD); absence of brackets at a point indicates the SD fell within the area occupied by the point. Note the different ordinate scales for each monkey.

With nFR schedules, deliveries are not directly fixed to the number of responses, because the number of responses per delivery can vary. When water was the alternative, the number of deliveries of 16% decreased as the nFR value became larger for three monkeys, or stayed relatively stable until decreasing at the highest nFR values for the remaining two monkeys (Fig. 3, left panels). However, when 8% was the alternative, deliveries of 16% ethanol tended to increase as nFR size reached the higher values, and then decrease as nFR size was increased still further (Fig. 3, right panels). Deliveries of 0 and 8% generally declined with increases in the schedule requirement.

**Figure 4:**
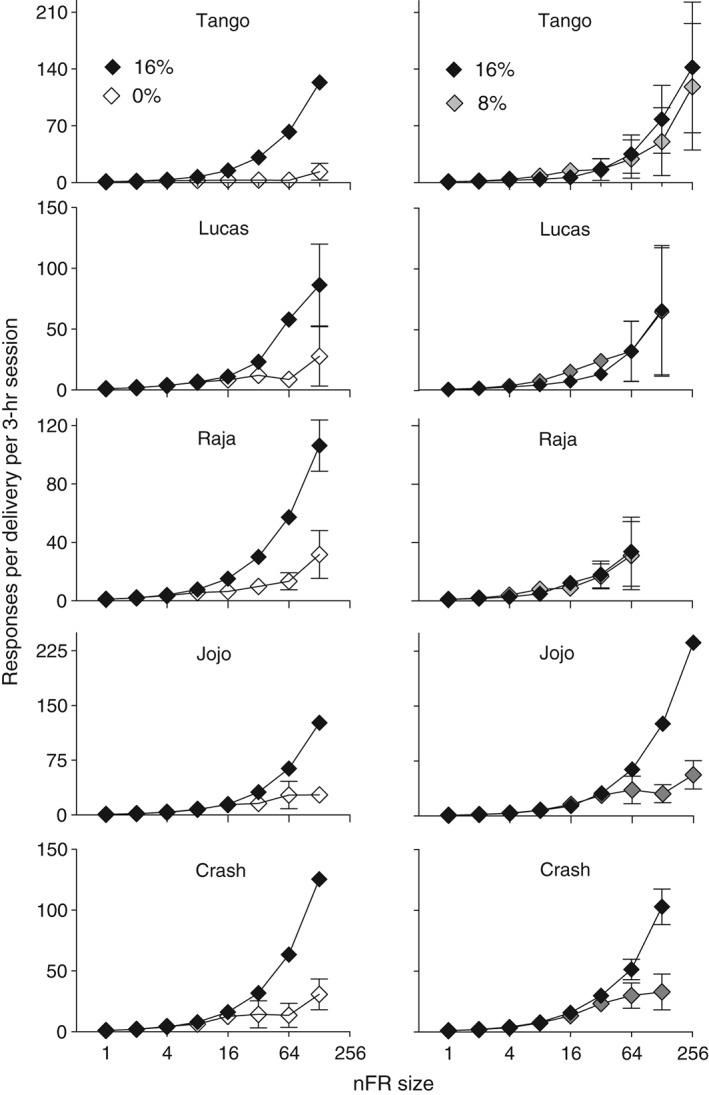
Responses per delivery as a function of nFR size for five monkeys. Panels on the left are for 16 and 0% ethanol (w/v) and panels on the right are for 16 and 8%. Each point is a mean of six consecutive sessions of stable behavior. Brackets denote the standard deviation (SD); absence of brackets at a point indicates the SD fell within the area occupied by the point. Note the different ordinate scales for each monkey.

With nFR schedules the number of responses per delivery becomes a graded dependent variable. Figure 4 shows that with 16% ethanol the number of responses per delivery increased directly with nFR size until the highest schedule size was reached, where responses per delivery declined. Sixteen percent ethanol maintained more responses per delivery than did water, and for two subjects, maintained more responses per delivery than 8% ethanol. For a third subject more responses per delivery were maintained by 16 than by 8% ethanol, but the bars showing standard errors of the means overlapped for these concentrations. At the highest ratio value that was studied with both pairs, the differences between 16 and 0% were greater than between 16 and 8%, though with monkey JoJo the difference was less than with the other monkeys.

**Figure 5:**
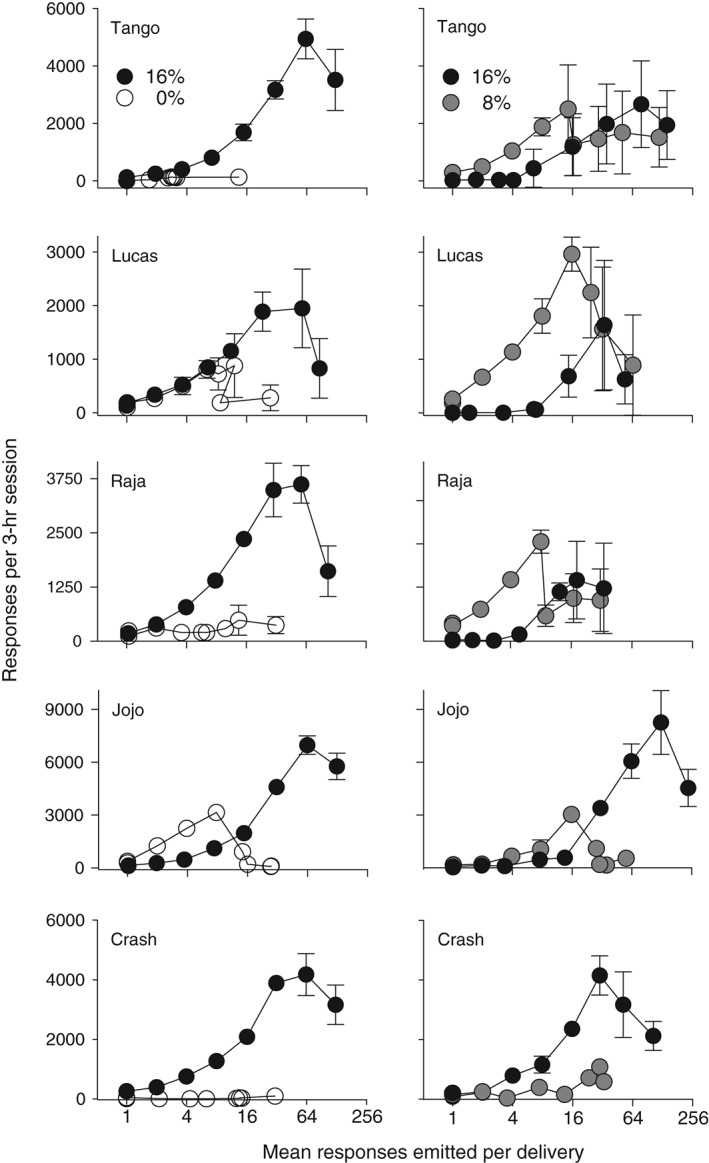
Response rates, as concurrent nFR schedule size increased, are plotted in relation to the dependent variable of responses emitted per delivery. The responses per session are the same as in Figure 1. However, the abscissa is different: It shows the mean number of responses per delivery. Panels on the left are for 16 and 0% ethanol (w/v) and panels on the right are for 16 and 8%. Each point is a mean of six consecutive sessions of stable behavior. Disconnected points at FR 1 represent redetermined values. Brackets denote the standard deviation (SD); absence of brackets at a point indicates the SD fell within the area occupied by the point. Note the different ordinate scales for each monkey.

Plotting total responses per 3‐hr session in relation to responses emitted per delivery (or price paid) highlights the differences between the liquids in maintaining behavior (Fig. 5). Responses per delivery tended to be substantially less with water and terminated at lower values than with 16% ethanol. When 16 and 8% were available, responses per delivery were initially higher with 8% ethanol. However, with increases in schedule size responses emitted per delivery for 16% became greater.

**Figure 6:**
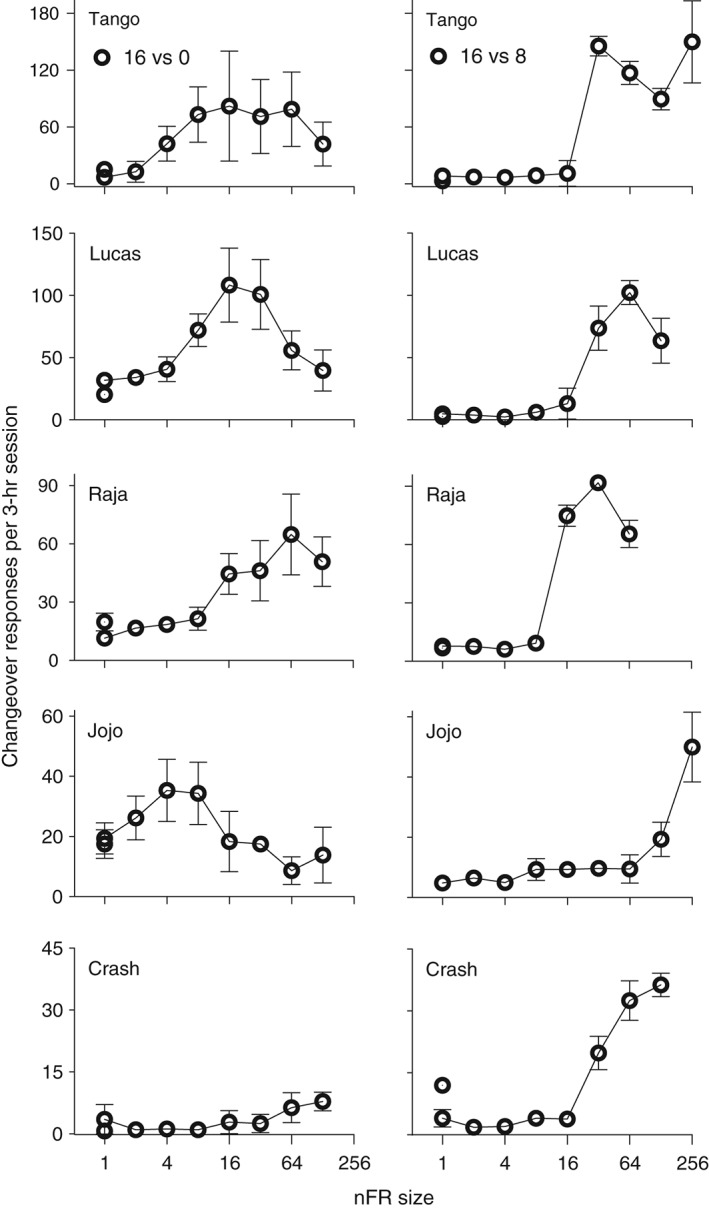
Changeover responses as a function of nFR size for five monkeys. Panels on the left are for 16 and 0% ethanol (w/v) and panels on the right are for 16 and 8%. Each point is a mean of six consecutive sessions of stable behavior. Disconnected points at FR 1 represent redetermined values. Brackets denote the standard deviation (SD); absence of brackets at a point indicates the SD fell within the area occupied by the point. Note the different ordinate scales for each monkey.

Changeover responses were a function of nFR size and liquid pair. When 16 and 0% ethanol were present, changeover responses were generally an inverted U‐shaped function of schedule size (Fig. 6, left panels). When 16 and 8% were available, changeover responses were initially low and then increased with the schedule size. The maximum numbers of changeover responses were also greater with the 16‐8% pair. It should be noted that changeover responses increase the amount of ethanol delivered per response emitted.

**Figure 7:**
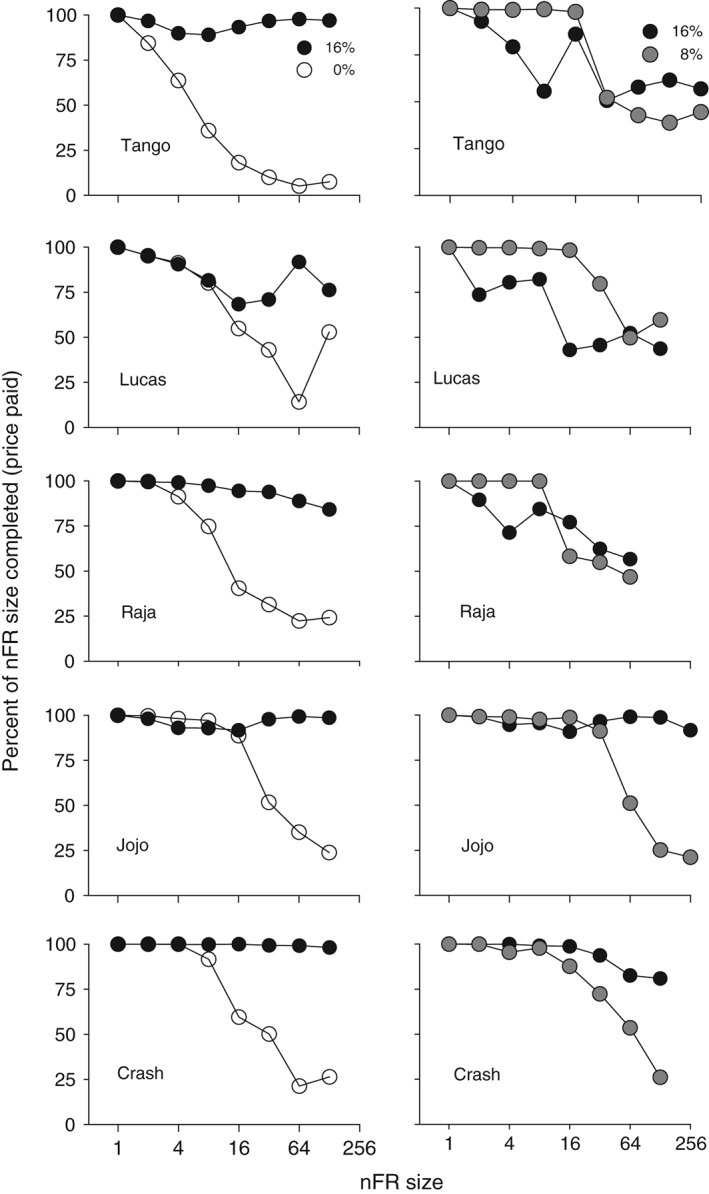
Percent of the nFR requirement completed for each liquid as a function of nFR size. The percent was calculated by dividing the number of responses by the total number of responses that would have been necessary in the absence of changeover responses. Panels on the left are for 16 and 0% ethanol (w/v) and panels on the right are for 16 and 8%. Each point is a mean of six consecutive sessions of stable behavior.

Use of a choice procedure and the measure of responses per delivery make possible calculation of derived measures. Figure 7 shows one such measure, the mean number of responses per delivery as a percent of the schedule size. Thus, for example, if reinforcer deliveries were collected under an nFR 64 schedule, after an average of 48 mouth‐contact responses on a spout, this value would show on Figure 7 as 75%. Other possible derivations are responses as a percent of theoretical maximum (actual responses divided by the product of schedule size and number of deliveries) and deliveries as a percent of theoretical maximum (number of actual responses divided by one half the schedule size, excluding FR 1).

**Figure 8:**
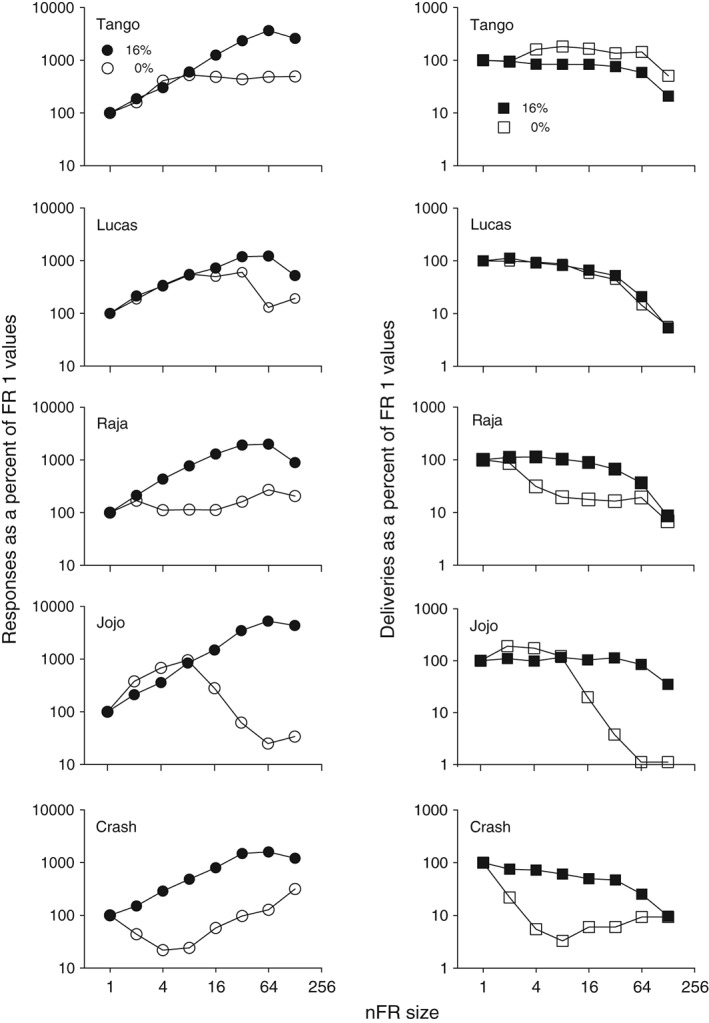
Mean percent responses (left panels) and mean percent deliveries (right panels) plotted as a percent of values obtained at FR 1, with 16% and 0% ethanol concurrently available. Each point is a mean of six consecutive sessions of stable behavior. Note that the range of the ordinates is from 10 to 10,000 for responses and from 1 to 1,000 for deliveries.

The relative persistence of behavior has been proposed as a measure of relative reinforcing effects (for a summary, see Meisch, 2000). Figure 8 illustrates responding and deliveries across nFR values as a percent of FR 1 values. When responses are the dependent variable (left panels), a clear separation emerges between responses reinforced with 16% ethanol and responses reinforced with the water vehicle. In contrast the number of deliveries as a percent of FR 1 values (right panels) shows less separation and in one case (Tango) a reversal.

## Discussion

### Responding

Choice under the nFR schedules was a function of both the pairs of liquids available and the number of responses required on the schedule. When 16% and 0% ethanol were present, the differences in rates of responding maintained by the two liquids increased with schedule size until overall rates declined (Fig. 1, left panels). The higher rates maintained by 16% relative to the water vehicle demonstrate that 16% served as a reinforcer. The inverted U‐shaped function between response rate and schedule size is similar to that seen when ethanol serves as a reinforcer for rats and FR size is varied (Meisch & Thompson, 1973). When the pair of liquids was 16 and 8% ethanol, relative response rates varied as a function of schedule size. Responding maintained by 16% increased as nFR size increased. Initially, responding maintained by 8% exceeded that maintained by 16%. However, as nFR size was increased all five monkeys showed an increase in relative responding for 16%, and for four of the monkeys responses for 16% ethanol were greater than for 8% ethanol. When the nFR size was decreased, preference reverted back to 8% for these four monkeys. For three monkeys (Tango, Lucas, and Raja), responding reinforced by 16% was suppressed when 8% ethanol was present (cf. left and right panels of Fig. 1). Generally the higher the intake was of 8%, the lower the intake of 16%. Thus, in behavioral economic terms, 8% served as an economic substitute.

### Preference Reversal

Preference reversals are of interest in part because they are contrary to the classical economic theory that preferences should remain constant (Kagel, Battalio & Green, 1995). Many studies have noted a reversal of preference from “sooner–smaller” reinforcers to “later–larger” reinforcers, when the delay is increased between the choice point and receipt of the reinforcer (e.g., Huskinson & Anderson, 2013). Preference reversals also have been seen with FR schedules. Human cigarette smokers given a choice between money and cigarettes selected money at low FR values and cigarettes at higher FR values (Bickel & Madden, 1999). These findings were confirmed in a second study with humans, where participants were given a choice between money and cigarettes (Johnson & Bickel, 2006). A possible explanation for these findings is that they may reflect a crossing of demand curves when each commodity is studied separately (Bickel & Madden, 1999; Johnson & Bickel, 2006). In a behavioral economic study of ethanol self‐administration, four monkeys preferred the water vehicle to 32% (w/v) ethanol at FR 4 (Williams & Woods, 2000). However, as FR size increased, responding maintained by 32% ethanol increased and responding maintained by water decreased until at FR 64 there was a clear preference for 32%. Similarly in the present study the monkey JoJo preferred the water vehicle to 16% ethanol at FR 1 (Figs. 1 and 3, left panels). Increases in the nFR size resulted in a preference for 16% at nFR 16 that was maintained across subsequent increases in nFR size.

Under FR schedules preference reversals have been observed with rhesus monkeys in studies of oral drug self‐administration (Meisch & Lemaire, 1990; Meisch, Stewart, & Wang, 1996; Stewart, Wang, Bass & Meisch, 2002). However, these reversals were obtained with one (Meisch, Stewart, & Wang, 1996) or two (Meisch & Lemaire, 1990; Stewart, Wang, Bass & Meisch, 2002) monkeys. In the present study a reversal in preference between 8 and 16% ethanol was seen with four of five monkeys when nFR schedules were used (see Fig. 1, right panels for subjects Tango, Raja and JoJo; Fig. 3, right panel for Crash). The fifth monkey showed a marked increase in intake of 16% alcohol, but not a clear reversal. Preference shifted from 8 to 16% ethanol as the nFR size increased, and the preference reverted to 8% when the nFR size decreased. When preference shifted, it usually did so immediately after a change in ratio size. The use of progressive‐ratio schedules may allow a determination of whether preference will change within sessions as the ratio size increases.

The present findings extend the results obtained with human participants choosing between cigarettes and money to rhesus monkeys, nonindependent ratio schedules, and choice between different amounts of the same reinforcer (alcohol). The present results support the idea that ratio size, or cost, can be a determinant of relative reinforcing effects (Bickel & Madden, 1999; Johnson & Bickel, 2006), and emphasize a point made by Bickel and Madden, that a determination of relative reinforcing effects at only one ratio size may lead to a conclusion that is not supported when other ratio sizes are studied.

#### Responses per delivery

With standard FR schedules the relation between responding and liquid deliveries is fixed. However, with nonindependent FR schedules, the number of responses per delivery becomes a graded dependent variable and is a potential measure of relative reinforcing effects. The differences in responses per delivery between 16% ethanol and the other liquid were greater when the other liquid was 0% rather than 8% ethanol (cf. left and right panels of Fig. 3). The reinforcing effects of 8% (vs. 16%) and 0% (vs. 16%) can be compared across the blocks of sessions. When concurrently available with 16% ethanol, at schedule sizes above nFR 16 the number of responses per delivery for 8% was greater than for 0%. The dependent variable of responses per delivery may be a more graded measure of relative reinforcing effects than relative response rates recorded under independent concurrent FR FR schedules (Meisch & Gomez, 2013). To use terminology from behavioral economics, the subject can select the relative price paid for each reinforcer. In contrast, price has usually been an independent variable in operant studies of behavioral economics (Hursh, Madden, Spiga, DeLeon & Francisco, 2013).

#### Changeover responses

Changeover responses are greater under nFR schedules than under independent ratio schedules (MacDonall, 1988; Meisch & Gomez, 2013; Shull & Pliskoff, 1971). Similarly, changeover responses are greater under concurrent interval schedules when simultaneous rather than sequential progress toward schedule completion is possible (Shull & Pliskoff, 1971). In the present study changeover responses were a function of both schedule size and liquid pair. Changeover responses increased with schedule size when the liquid pair was 16 and 8%, but less so with 16 and 0% (cf. left and right panels of Fig. 6). These findings are consistent with an analysis in terms of optimizing reinforcer intake per response or per time interval. If the governing principle is optimizing reinforcer intake per response or time interval, then changeover responses should be greatest when schedule sizes (Davison & McCarthy, 1988) or reinforcer effects are equal. Also, greater increases in changeover responses would be expected when schedule size increases, since the effects of changeover responses would be larger. To summarize, more changeovers occur (a) at larger schedule sizes, (b) with smaller differences in concurrent reinforcer magnitudes, and (c) with smaller differences in concurrent schedule sizes. Changeover responses are increased with schedules in which there is simultaneous rather than sequential progress toward schedule completion, independent of whether the schedules are ratio or interval schedules. Our findings and those of others support this analysis (e.g., Davison & McCarthy, 1988).

#### Choice between reinforcers

Nonindependent ratio schedules offer additional means of assessing choice due to increases in changeover responses. Unlike interval schedules, pausing does not result in fewer responses per reinforcer, and exclusive preferences do not develop. Thomsen, Barret, Negus, and Caine (2013) have emphasized the importance of choice in drug self‐administration studies. Nonindependent ratio schedules make possible concurrent responding that could be maintained by a nondrug reinforcer, in conjunction with responding maintained by a drug reinforcer. Importantly, responding maintained by each reinforcer is interspersed over the same intervals instead of separate blocks. By adjusting the magnitude of the second reinforcer, equal response rates could be established. A relatively selective treatment effect would be detected by any manipulation that resulted in greater changes in drug‐reinforced responding than in responding maintained by the second reinforcer. Also, changes in responses per reinforcer delivery can also aid in measuring the relative outcomes of treatments such as other drugs or lesions. With nonindependent schedules the second operant will maintain higher changeover rates than would occur under independent schedules. Investigators (Banks, Hutsell, Blough, Poklis, & Negus, 2015; Winsauer, Moerschbaecher, & Roussell, 2008) have noted the relevance of a second operant in assessing the clinical potential of candidate treatment drugs as the second operant can aid in detecting a drug's nonselective actions.

#### Classification of schedules

Evaluation of the significance of nonindependent schedules is aided by an alternative view of how concurrent schedules can be classified. Shull and Pliskoff (1971) suggested that nonindependent FR and VR schedules “simulate” concurrent FI and VI schedules, because responding on one schedule increases the probability of reinforcement for responding on the other schedule. However, the nonindependent ratio schedules are more than simulations, for, as MacDonall (1988) noted, concurrent nonindependent VR VR schedules are the formal and functional equivalent of concurrent VI VI schedules. Concurrent VI VI and FI FI schedules have frequently been classified as “independent” schedules since the program for each schedule operates independently of the other schedule (Davison & McCarthy, 1988). A different way to classify concurrent schedules is based on their effects on behavior rather than how they are programmed. One such alternative classification would be to group schedules on the basis of whether there is simultaneous (nonindependent) or sequential (independent) progress toward schedule completion, rather than on whether the schedules are programmed independently. From this perspective, what have been termed concurrent independent VI VI schedules would instead be termed concurrent nonindependent VI VI schedules, because there can be simultaneous progress toward completion of both schedule requirements. Such a classification emphasizes previously under‐appreciated relations between ratio and interval schedules, namely, the formal and functional equivalence of nonindependent ratio schedules with concurrent interval schedules, as both permit simultaneous progress toward completion of the schedule requirements (cf. MacDonall, 1988).

#### Conclusions

Responding under nFR schedules is sensitive to differences in the effects of concurrent reinforcers. In the present study, over a broad range of schedule sizes differences in response rates maintained by different reinforcers increased with schedule size. At nFR values above 32 the differences were graded, with 16% > 8% > 0% ethanol. Importantly, the changes in preference for 16% ethanol across changes in schedule size show that the size of the schedule is a determinant of relative reinforcing effects. The graded dependent variable of responses per reinforcer is an additional measure of relative reinforcing effects. The nonindependent‐ratio schedules provide an additional means of assessing choice due to the increase in the number of changeover responses, an important dependent variable with these schedules. These ratio schedules avoid a problem that can develop with interval schedules when response rates are low, namely, reinforcement of the first response after an extended pause, because with ratio schedules low response rates do not alter the ratio requirement. A second operant can provide a measure of selective effects of candidate medications by use of an alternative, but equally preferred nondrug reinforcer. Most studies of choice have used interval schedules since they increase contact with both response alternatives. The study of nonindependent ratio schedules should increase our understanding of choice because they are similar in important ways with concurrent interval schedules, the fundamental difference being that schedule completion is based on responses rather than time. The differences between behavior observed under independent and nonindependent ratio schedules emphasize the fundamental distinction between concurrent schedules that permit simultaneous versus sequential progress toward schedule completion. When only sequential progress is possible, the usual result is an exclusive preference.

## References

[jeab215-bib-0001] Banks, M. L. , Hutsell, B. A. , Blough, B. E. , Poklis, J. L. , & Negus, S. S. (2015). Preclinical assessment of lisdexamfetamine as an agonist medication candidate for cocaine addiction: Effects in rhesus monkeys trained to discriminate cocaine or to self‐administer cocaine in a cocaine versus food choice procedure. International Journal of Neuropsychopharmacology, 18, 1–10. 10.1093/ijnp/pyv009 PMC4458439 25618405

[jeab215-bib-0002] Bickel, W. K. , & Madden, G. J. (1999). A comparison of measures of relative reinforcing efficacy and behavioral economics: Cigarettes and money in smokers. Behavioural Pharmacology, 10, 627–637. 10780504 10.1097/00008877-199911000-00009

[jeab215-bib-0003] Carr, K. D. (2002). Augmentation of drug reward by chronic food restriction: Behavioral evidence and underlying mechanisms. Physiology & Behavior, 76, 353–364. 12117572 10.1016/s0031-9384(02)00759-x

[jeab215-bib-0004] Carroll, M. E. , & Meisch R. A. (1984). Enhanced drug‐reinforced behavior due to food deprivation In Thompson T., Dews P. B., & Barrett J. E. (Eds.) Advances in behavioral pharmacology (Vol. 4, pp. 47–88). New York: Academic Press.

[jeab215-bib-0005] Davison, M. , & McCarthy, D. (1988). The matching law: A research review. Hillsdale, NJ: Erlbaum.

[jeab215-bib-0006] Gieske, D. (1978). Integrated drinking device for monkeys (Tech. Rep. PR‐78‐1). Minneapolis: University of Minnesota, Department of Psychiatry.

[jeab215-bib-0007] Gomez, T. H. , & Meisch, R. A. (2003). Relation between choice of ethanol concentration and response rates under progressive‐ and fixed‐ratio schedules: Studies with rhesus monkeys. Psychopharmacology, 170, 1–8. 12802578 10.1007/s00213-003-1489-8

[jeab215-bib-0008] Henningfield, J. E. , & Meisch, R. A. (1976). Drinking device for rhesus monkeys. Pharmacology, Biochemistry, & Behavior, 4, 609–610. 10.1016/0091-3057(76)90204-5 821063

[jeab215-bib-0009] Henningfield, J. E. , & Meisch, R. A. (1978). Ethanol drinking by rhesus monkeys as a function of concentration. Psychopharmacology, 57, 133–136. 418445 10.1007/BF00426877

[jeab215-bib-0011] Hursh, S. R. , Madden, G. J. , Spiga, R. , DeLeon, I. G. , & Francisco, M. T. (2013). The translational utility of behavioral economics: The experimental analysis of consumption and choice In Madden G. J., Dube W. V., Hackenberg T. D., Hanley G. P., & Lattal K. A. (Eds.), APA Handbook of Behavior Analysis (Vol. 2, pp. 191–224). Washington, DC: American Psychological Association.

[jeab215-bib-0012] Huskinson, S. L. , & Anderson, K. G. (2013). Effects of different fixed‐ratio requirements on delay discounting in rats. Behavioural Processes, 100, 18–22. 23891790 10.1016/j.beproc.2013.07.013

[jeab215-bib-0013] Institute for Laboratory Animal Research . (2011). Guide for the care and use of laboratory animals (8th ed.). Washington, DC: National Academies Press.

[jeab215-bib-0014] Johnson, M. W. , & Bickel, W. K. (2006). Replacing relative reinforcing efficacy with behavioral economic demand curves. Journal of the Experimental Analysis of Behavior, 85, 73–93. 16602377 10.1901/jeab.2006.102-04 PMC1397796

[jeab215-bib-0015] Kagel, J. H. , Battalio, R. C. , & Green, L. (1995). Economic choice theory: An experimental analysis of animal behavior. Cambridge: Cambridge University Press.

[jeab215-bib-0016] Kemnitz, J. W. (2011). Calorie restriction and aging in nonhuman primates. ILAR Journal, 52, 66–77. 21411859 10.1093/ilar.52.1.66 PMC3278796

[jeab215-bib-0017] Kliner, D. J. , & Meisch, R. A. (1989). Oral pentobarbital intake in rhesus monkeys: Effects of drug concentration under conditions of food deprivation and satiation. Pharmacology, Biochemistry, & Behavior, 32, 347–354. 10.1016/0091-3057(89)90253-0 2734345

[jeab215-bib-0018] MacDonall, J. S. (1988). Concurrent variable‐ratio schedules: Implications for the generalized matching law. Journal of the Experimental Analysis of Behavior, 50, 55–64. 16812549 10.1901/jeab.1988.50-55 PMC1338840

[jeab215-bib-0019] MacDonall, J. S. (1998). Run length, visit duration, and reinforcers per visit in concurrent performance. Journal of the Experimental Analysis of Behavior, 69, 275–293. 16812878 10.1901/jeab.1998.69-275 PMC1284664

[jeab215-bib-0020] MacDonall, J. S. (1999). A local model of concurrent performance. Journal of the Experimental Analysis of Behavior, 71, 57–74. 16812892 10.1901/jeab.1999.71-57 PMC1284694

[jeab215-bib-0021] Macenski, M. J. , & Meisch, R. A. (1992). Ethanol‐reinforced responding of naive rhesus monkeys: Acquisition without induction procedures. Alcohol, 9, 547–554. 1472312 10.1016/0741-8329(92)90095-r

[jeab215-bib-0022] Mattison, J. A. , Roth, G. S. , Lane, M. A. , & Ingram, D. K. (2007). Dietary restriction in aging nonhuman primates. Interdisciplinary Topics in Gerontology and Geriatrics, 35, 137–158. 10.1159/000096560 17063037

[jeab215-bib-0023] Meisch, R.A. (2000). Relative persistence of behavior: A fundamental measure of relative reinforcing effects. Experimental and Clinical Psychopharmacology, 8, 333–349. 10975621 10.1037//1064-1297.8.3.333

[jeab215-bib-0024] Meisch, R. A. , & Gomez, T. H. (2013). Drug self‐administration studies: A novel reinforcement schedule enhances choice. Behavioural Pharmacology, 24, 155–163. 23549451 10.1097/FBP.0b013e32836104cd

[jeab215-bib-0025] Meisch, R. A. , & Lemaire, G. A. (1988). Oral self‐administration of pentobarbital by rhesus monkeys: Relative reinforcing effects under concurrent fixed‐ratio schedules. Journal of the Experimental Analysis of Behavior, 50, 75–86. 3171474 10.1901/jeab.1988.50-75 PMC1338842

[jeab215-bib-0026] Meisch, R. A. , & Lemaire, G. A. (1990). Reinforcing effects of a pentobarbital–ethanol combination relative to each drug alone. Pharmacology, Biochemistry, & Behavior, 35 443–450. 10.1016/0091-3057(90)90182-h 2320653

[jeab215-bib-0027] Meisch, R. A. , & Lemaire, G. A. (1991). Effects of feeding condition on orally delivered ethanol as a reinforcer for rhesus monkeys. Alcohol, 8, 55–63. 2006986 10.1016/0741-8329(91)91264-3

[jeab215-bib-0028] Meisch, R. A. , & Spiga, R. (1998). Matching under nonindependent variable‐ratio schedules of drug reinforcement. Journal of the Experimental Analysis of Behavior, 70, 23–34. 9684343 10.1901/jeab.1998.70-23 PMC1284667

[jeab215-bib-0029] Meisch, R. A. , Stewart, R. B. , & Wang, N.‐S. (1996). Orally delivered methadone as a reinforcer for rhesus monkeys: The relationship between drug concentration and choice. Pharmacology, Biochemistry, & Behavior, 54, 547–554. 10.1016/0091-3057(95)02214-7 8743628

[jeab215-bib-0030] Meisch, R. A. , & Thompson, T. (1973). Ethanol as a reinforcer: Effects of fixed‐ratio size and food deprivation. Psychopharmacologia, 28, 171–183. 4694625 10.1007/BF00421402

[jeab215-bib-0031] Shull, R. L. , & Pliskoff, S. S. (1971). Changeover behavior under pairs of fixed‐ratio and variable‐ratio schedules of reinforcement. Journal of the Experimental Analysis of Behavior, 16, 75–79. 16811536 10.1901/jeab.1971.16-75 PMC1333825

[jeab215-bib-0032] Stewart, R. B. , Bass A. A. , Wang, N.‐S. , & Meisch, R. A. (1996). Ethanol as a reinforcer in normal weight rhesus monkeys. Dose–response functions. Alcohol, 13, 341–346. 8836321 10.1016/0741-8329(96)00004-3

[jeab215-bib-0033] Stewart, R. B. , Wang, N. S. , Bass, A. A. , & Meisch, R. A. (2002). Relative reinforcing effects of different oral ethanol doses in rhesus monkeys. Journal of the Experimental Analysis of Behavior, 77, 49–64. 11831783 10.1901/jeab.2002.77-49 PMC1284847

[jeab215-bib-0034] Thomsen, M. , Barrett, A. C. , Negus, S. S. , & Caine, S. B. (2013). Cocaine versus food choice procedure in rats: Environmental manipulations and effects of amphetamine. Journal of the Experimental Analysis of Behavior, 99, 211–233. 23319458 10.1002/jeab.15 PMC3893350

[jeab215-bib-0035] Williams, K. L. , & Woods, J. H. (2000). A behavioral economic analysis of concurrent ethanol‐ and water‐reinforced responding in different preference conditions. Alcoholism: Clinical and Experimental Research, 24, 980–986. 10924000

[jeab215-bib-0036] Winsauer, P. J. , Moerschbaecher, J. M. , & Roussell, A. M. (2008). Differential antagonism of cocaine self‐administration and cocaine‐induced disruptions of learning by haloperidol in rhesus monkeys. Journal of the Experimental Analysis of Behavior, 89, 225–246. 18422020 10.1901/jeab.2008.89-225 PMC2251325

